# Hexaaqua­gallium(III) trinitrate trihydrate

**DOI:** 10.1107/S1600536809028086

**Published:** 2009-07-22

**Authors:** Arthur D. Hendsbee, Cory C. Pye, Jason D. Masuda

**Affiliations:** a Department of Chemistry, Saint Mary’s University, Halifax, Nova Scotia, Canada B3H 3C3

## Abstract

The title compound, [Ga(H_2_O)_6_](NO_3_)_3_·3H_2_O, is isostructural to other known *M* ^III^ nitrate hydrates (*M* = Al, Cr, Fe). The structure contains two distinct octa­hedral Ga(OH_2_)_6_ units (each of 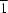 symmetry) which are involved in inter­molecular hydrogen bonding with the three nitrate anions and three water mol­ecules within the asymmetric unit.

## Related literature

For the the aluminium analogue, see: Lazar, Ribár, Divjaković & Mészáros (1991 ▶). For the chromium analogue, see: Lazar, Ribár & Prelesnik (1991 ▶). For the iron analogue, see: Hair & Beattie (1977 ▶). For ionic radii, see: Shannon & Prewitt (1969 ▶). Gallium nitrate, used in the preparation, easily forms supersaturated solutions, see: Rudolph *et al.* (2002 ▶), and hence the sample was cooled to 248 K and a seed crystal was introduced to initiate crystallization.
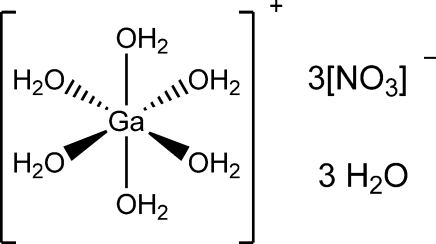


## Experimental

### 

#### Crystal data

[Ga(H_2_O)_6_](NO_3_)_3_·3H_2_O
*M* *_r_* = 417.89
Monoclinic, 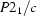

*a* = 13.9609 (6) Å
*b* = 9.6498 (5) Å
*c* = 10.9743 (5) Å
β = 95.448 (1)°
*V* = 1471.78 (12) Å^3^
*Z* = 4
Mo *K*α radiation
μ = 1.97 mm^−1^
*T* = 296 K
0.40 × 0.34 × 0.29 mm

#### Data collection

Bruker APEXII CCD diffractometer
Absorption correction: multi-scan (*SADABS*; Bruker, 2008 ▶) *T*_min_ = 0.479, *T*_max_ = 0.564
10587 measured reflections
3037 independent reflections
2509 reflections with *I* > 2σ(*I*)
*R*_int_ = 0.015

#### Refinement

*R*[*F* ^2^ > 2σ(*F* ^2^)] = 0.021
*wR*(*F* ^2^) = 0.058
*S* = 1.05
3037 reflections
274 parameters
18 restraints
H atoms treated by a mixture of independent and constrained refinement
Δρ_max_ = 0.48 e Å^−3^
Δρ_min_ = −0.33 e Å^−3^

### 

Data collection: *APEX2* (Bruker, 2008 ▶); cell refinement: *SAINT* (Bruker, 2008 ▶); data reduction: *SAINT*; program(s) used to solve structure: *SHELXS97* (Sheldrick, 2008 ▶); program(s) used to refine structure: *SHELXL97* (Sheldrick, 2008 ▶); molecular graphics: *ORTEP-3 for Windows* (Farrugia, 1997 ▶); software used to prepare material for publication: *SHELXTL* (Sheldrick, 2008 ▶).

## Supplementary Material

Crystal structure: contains datablocks I, global. DOI: 10.1107/S1600536809028086/mg2076sup1.cif

Structure factors: contains datablocks I. DOI: 10.1107/S1600536809028086/mg2076Isup2.hkl

Additional supplementary materials:  crystallographic information; 3D view; checkCIF report

## Figures and Tables

**Table 1:** Hydrogen-bond geometry (Å, °)

*D*—H⋯*A*	*D*—H	H⋯*A*	*D*⋯*A*	*D*—H⋯*A*
O18—H18⋯O8	0.801 (16)	2.26 (2)	2.9348 (18)	142 (2)
O16—H14⋯O18	0.825 (15)	2.072 (15)	2.8732 (19)	163.8 (19)
O5—H10⋯O7	0.823 (16)	1.908 (17)	2.7052 (17)	163 (2)
O1—H1⋯O16	0.814 (15)	1.846 (16)	2.6474 (16)	168 (2)
O4—H7⋯O14	0.809 (15)	1.833 (15)	2.6399 (15)	175 (2)
O5—H9⋯O17	0.810 (16)	1.869 (16)	2.676 (2)	174 (2)
O18—H17⋯O14	0.816 (16)	2.082 (17)	2.8729 (18)	163 (2)
O3—H6⋯O15^i^	0.814 (15)	1.903 (16)	2.7150 (16)	175 (2)
O1—H2⋯O10^i^	0.808 (15)	1.848 (16)	2.6545 (16)	175 (2)
O2—H4⋯O16^i^	0.790 (16)	1.901 (16)	2.6895 (18)	175 (2)
O4—H8⋯O17^ii^	0.821 (15)	1.816 (15)	2.6312 (16)	171 (2)
O17—H15⋯O9^ii^	0.808 (15)	1.977 (16)	2.7791 (19)	171 (2)
O3—H5⋯O13^iii^	0.792 (15)	1.961 (16)	2.7454 (16)	171 (2)
O6—H12⋯O12^iv^	0.796 (15)	1.926 (16)	2.7179 (16)	174 (2)
O16—H13⋯O18^v^	0.820 (16)	1.934 (16)	2.7525 (19)	177 (3)
O6—H11⋯O11^vi^	0.800 (15)	1.895 (16)	2.6938 (17)	176 (2)
O2—H3⋯O8^vii^	0.794 (15)	1.943 (16)	2.7269 (17)	169 (2)
O17—H16⋯O7^viii^	0.802 (16)	2.026 (18)	2.7675 (18)	154 (2)

Symmetry codes: (i) 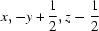; (ii) 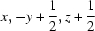; (iii) 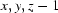; (iv) 
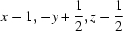
; (v) 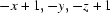; (vi) 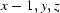; (vii) 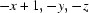; (viii) 
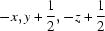
.

**Fig. 1.**
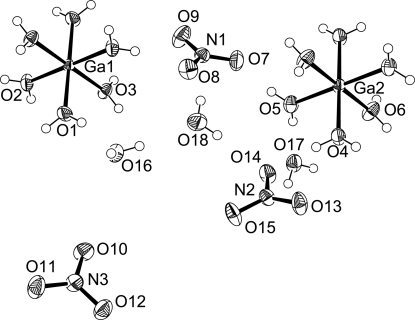
[Ga(H2O)6](NO3).3H2O with thermal ellipsoids shown at 50% probability level.

**Fig. 2.**
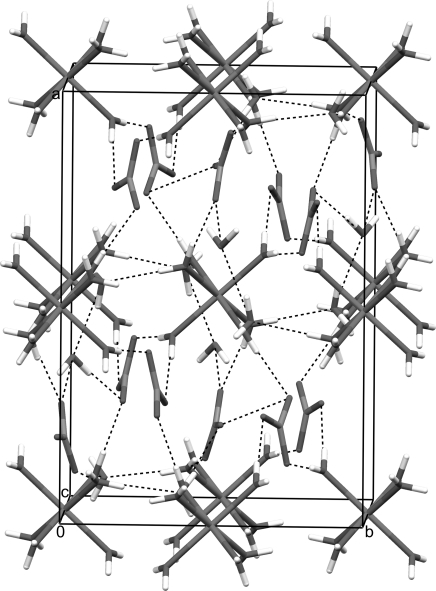
Packing diagram viewed down the c-axis with hydrogen bonds indicated by dashed lines.

## supplementary crystallographic information

### Comment

The title compound is isostructural with [Al(H_2_O)_6_](NO_3_)_3_.3H_2_O (Lazar, Ribár, Divjaković & Mészáros, 1991), [Cr(H_2_O)_6_](NO_3_)_3_.3H_2_O (Lazar, Ribár & Prelesnik, 1991) and [Fe(H_2_O)_6_](NO_3_)_3_.3H_2_O (Hair & Beattie, 1977). Its unit cell volume is almost identical to that of the chromium derivative (1473.87 (17) Å^3^) and intermediate between those of the aluminum (1448.9 (4) Å^3^) and iron derivatives 1489.8 (2) Å^3^), consistent with the values of ionic radii (Ga^3+^, 0.760 Å; Cr^3+^, 0.755 Å; Al^3+^, 0.670 Å; Fe^3+^, 0.785 Å) (Shannon & Prewitt, 1969). On each of the octahedral units there are two symmetry-related water molecules which hydrogen bond to two NO_3_^-^ anions. The remaining metal-bound water molecules participate in intermolecular hydrogen bonding with one NO_3_^-^ anion and one of the interstitial H_2_O molecules.

### Experimental

The title compound was prepared by dissolving 5 grams of gallium(III) nitrate hydrate (Aldrich Chemical Company) in a minimum of H_2_O (approximately 7 ml) and adding three drops of concentrated nitric acid to suppress hydrolysis. Because gallium nitrate easily forms supersaturated solutions (Rudolph *et al.*, 2002), the sample was cooled to 248 K and a seed crystal was introduced to initiate crystallization. A suitable crystal was sealed in a glass capillary to prevent water loss from this hygroscopic material.

### Refinement

The H atoms were found in the electron difference map and O-H distances fixed to 0.82 Å.

### Crystal data

**Table:**

[Ga(H_2_O)_6_](NO_3_)_3_·3H_2_O	*F*(000) = 856
*M**_r_* = 417.89	*D*_x_ = 1.886 Mg m^−^^3^
Monoclinic, *P*2_1_/*c*	Mo *K*α radiation, λ = 0.71073 Å
Hall symbol: -P 2ybc	Cell parameters from 5403 reflections
*a* = 13.9609 (6) Å	θ = 2.3–28.3°
*b* = 9.6498 (5) Å	µ = 1.97 mm^−^^1^
*c* = 10.9743 (5) Å	*T* = 296 K
β = 95.448 (1)°	Irregular, colourless
*V* = 1471.78 (12) Å^3^	0.40 × 0.34 × 0.29 mm
*Z* = 4	

### Data collection

**Table:**

Bruker APEXII CCD diffractometer	3037 independent reflections
Radiation source: fine-focus sealed tube	2509 reflections with *I* > 2σ(*I*)
graphite	*R*_int_ = 0.015
φ and ω scans	θ_max_ = 26.5°, θ_min_ = 2.6°
Absorption correction: multi-scan (*SADABS*; Bruker, 2008)	*h* = −17→17
*T*_min_ = 0.479, *T*_max_ = 0.564	*k* = −12→10
10587 measured reflections	*l* = −13→13

### Refinement

**Table:**

Refinement on *F*^2^	Primary atom site location: structure-invariant direct methods
Least-squares matrix: full	Secondary atom site location: difference Fourier map
*R*[*F*^2^ > 2σ(*F*^2^)] = 0.021	Hydrogen site location: inferred from neighbouring sites
*wR*(*F*^2^) = 0.058	H atoms treated by a mixture of independent and constrained refinement
*S* = 1.05	*w* = 1/[σ^2^(*F*_o_^2^) + (0.0301*P*)^2^ + 0.4119*P*] where *P* = (*F*_o_^2^ + 2*F*_c_^2^)/3
3037 reflections	(Δ/σ)_max_ < 0.001
274 parameters	Δρ_max_ = 0.48 e Å^−^^3^
18 restraints	Δρ_min_ = −0.33 e Å^−^^3^

### Special details

**Table:**

Geometry. All esds (except the esd in the dihedral angle between two l.s. planes) are estimated using the full covariance matrix. The cell esds are taken into account individually in the estimation of esds in distances, angles and torsion angles; correlations between esds in cell parameters are only used when they are defined by crystal symmetry. An approximate (isotropic) treatment of cell esds is used for estimating esds involving l.s. planes.
Refinement. Refinement of *F*^2^ against ALL reflections. The weighted *R*-factor *wR* and goodness of fit *S* are based on *F*^2^, conventional *R*-factors *R* are based on *F*, with *F* set to zero for negative *F*^2^. The threshold expression of *F*^2^ > σ(*F*^2^) is used only for calculating *R*-factors(gt) *etc*. and is not relevant to the choice of reflections for refinement. *R*-factors based on *F*^2^ are statistically about twice as large as those based on *F*, and *R*- factors based on ALL data will be even larger.

### Fractional atomic coordinates and isotropic or equivalent isotropic displacement parameters (Å^2^)

**Table:**

	*x*	*y*	*z*	*U*_iso_*/*U*_eq_	
Ga1	0.5000	0.0000	0.0000	0.01867 (8)	
Ga2	0.0000	0.0000	0.5000	0.02131 (8)	
N1	0.19260 (10)	0.00919 (13)	0.12573 (13)	0.0285 (3)	
N2	0.29851 (9)	0.18907 (14)	0.70878 (11)	0.0268 (3)	
N3	0.78610 (9)	0.28563 (14)	0.71869 (11)	0.0293 (3)	
O1	0.57018 (8)	0.08802 (13)	0.13967 (10)	0.0306 (3)	
H2	0.6187 (12)	0.132 (2)	0.1335 (19)	0.051 (6)*	
H1	0.5546 (14)	0.088 (2)	0.2093 (15)	0.051 (6)*	
O2	0.57327 (8)	0.10539 (13)	−0.11126 (10)	0.0294 (2)	
H4	0.5629 (16)	0.1854 (17)	−0.121 (2)	0.053 (7)*	
H3	0.6243 (12)	0.082 (2)	−0.1307 (18)	0.054 (6)*	
O3	0.40601 (8)	0.14903 (12)	−0.00574 (10)	0.0269 (2)	
H6	0.3964 (15)	0.190 (2)	0.0569 (16)	0.051 (6)*	
H5	0.3607 (13)	0.150 (2)	−0.0550 (17)	0.051 (6)*	
O4	0.07132 (8)	0.09427 (14)	0.63446 (10)	0.0327 (3)	
H8	0.0636 (14)	0.093 (2)	0.7077 (14)	0.043 (5)*	
H7	0.1244 (12)	0.121 (2)	0.6219 (19)	0.051 (6)*	
O5	0.07408 (9)	0.10908 (14)	0.39031 (10)	0.0339 (3)	
H10	0.0971 (15)	0.084 (2)	0.3275 (17)	0.065 (7)*	
H9	0.0633 (16)	0.1911 (17)	0.381 (2)	0.054 (7)*	
O6	−0.09607 (8)	0.14720 (14)	0.49563 (11)	0.0358 (3)	
H12	−0.1406 (13)	0.147 (2)	0.4446 (18)	0.054 (7)*	
H11	−0.1078 (16)	0.183 (2)	0.5582 (17)	0.055 (7)*	
O7	0.11323 (8)	0.04401 (15)	0.16045 (11)	0.0439 (3)	
O8	0.26475 (9)	−0.00369 (13)	0.19969 (12)	0.0430 (3)	
O9	0.19968 (11)	−0.01084 (15)	0.01616 (12)	0.0529 (4)	
O10	0.72635 (8)	0.25702 (13)	0.62972 (10)	0.0369 (3)	
O11	0.87278 (8)	0.26164 (15)	0.71226 (11)	0.0473 (3)	
O12	0.75836 (8)	0.33667 (15)	0.81328 (10)	0.0424 (3)	
O13	0.26276 (8)	0.16544 (14)	0.80530 (10)	0.0395 (3)	
O14	0.24653 (7)	0.18488 (13)	0.60783 (9)	0.0339 (3)	
O15	0.38523 (7)	0.21721 (14)	0.70780 (10)	0.0400 (3)	
O16	0.53516 (9)	0.12179 (13)	0.37069 (11)	0.0333 (3)	
H14	0.4793 (11)	0.108 (2)	0.3864 (17)	0.043 (6)*	
H13	0.5662 (15)	0.076 (2)	0.4232 (18)	0.063 (7)*	
O17	0.05170 (9)	0.38374 (14)	0.36978 (11)	0.0362 (3)	
H15	0.0906 (14)	0.427 (2)	0.4140 (19)	0.057 (7)*	
H16	0.0021 (13)	0.420 (2)	0.383 (2)	0.056 (7)*	
O18	0.35675 (10)	0.02473 (15)	0.45021 (13)	0.0410 (3)	
H17	0.3188 (14)	0.075 (2)	0.482 (2)	0.064 (8)*	
H18	0.3232 (17)	−0.016 (2)	0.399 (2)	0.062 (8)*	

### Atomic displacement parameters (Å^2^)

**Table:**

	*U*^11^	*U*^22^	*U*^33^	*U*^12^	*U*^13^	*U*^23^
Ga1	0.01679 (12)	0.02124 (13)	0.01796 (12)	0.00073 (8)	0.00156 (8)	−0.00138 (8)
Ga2	0.01777 (12)	0.02999 (15)	0.01606 (12)	−0.00007 (8)	0.00097 (8)	−0.00210 (8)
N1	0.0297 (7)	0.0278 (8)	0.0280 (7)	−0.0019 (5)	0.0029 (6)	0.0003 (5)
N2	0.0238 (6)	0.0302 (7)	0.0261 (6)	−0.0027 (5)	0.0002 (5)	0.0037 (5)
N3	0.0290 (7)	0.0308 (7)	0.0275 (7)	0.0044 (6)	−0.0005 (5)	−0.0004 (5)
O1	0.0271 (6)	0.0417 (7)	0.0227 (6)	−0.0094 (5)	0.0012 (4)	−0.0071 (5)
O2	0.0258 (6)	0.0284 (7)	0.0356 (6)	0.0032 (5)	0.0116 (5)	0.0065 (5)
O3	0.0245 (5)	0.0316 (6)	0.0240 (6)	0.0097 (5)	−0.0002 (4)	−0.0029 (5)
O4	0.0246 (6)	0.0536 (8)	0.0196 (6)	−0.0108 (5)	0.0018 (4)	−0.0078 (5)
O5	0.0395 (6)	0.0376 (8)	0.0265 (6)	−0.0047 (6)	0.0119 (5)	0.0013 (5)
O6	0.0314 (6)	0.0480 (8)	0.0269 (6)	0.0158 (5)	−0.0024 (5)	−0.0064 (5)
O7	0.0317 (6)	0.0576 (8)	0.0446 (7)	0.0009 (6)	0.0141 (5)	−0.0047 (6)
O8	0.0379 (7)	0.0536 (9)	0.0361 (7)	0.0013 (5)	−0.0045 (5)	0.0039 (5)
O9	0.0524 (9)	0.0741 (11)	0.0329 (7)	0.0144 (7)	0.0072 (6)	−0.0110 (6)
O10	0.0353 (6)	0.0431 (7)	0.0301 (6)	0.0072 (5)	−0.0080 (5)	−0.0057 (5)
O11	0.0271 (6)	0.0721 (10)	0.0422 (7)	0.0116 (6)	0.0006 (5)	−0.0133 (6)
O12	0.0339 (6)	0.0605 (9)	0.0324 (6)	0.0064 (6)	0.0018 (5)	−0.0149 (6)
O13	0.0301 (6)	0.0633 (9)	0.0255 (6)	−0.0049 (6)	0.0053 (5)	0.0088 (6)
O14	0.0262 (5)	0.0503 (7)	0.0242 (5)	−0.0054 (5)	−0.0023 (4)	0.0028 (5)
O15	0.0214 (5)	0.0629 (9)	0.0350 (6)	−0.0103 (5)	−0.0009 (4)	0.0128 (6)
O16	0.0336 (6)	0.0346 (7)	0.0323 (6)	0.0031 (5)	0.0066 (5)	0.0016 (5)
O17	0.0311 (6)	0.0496 (8)	0.0279 (6)	−0.0003 (6)	0.0024 (5)	−0.0019 (5)
O18	0.0383 (7)	0.0469 (8)	0.0365 (7)	−0.0027 (6)	−0.0027 (6)	−0.0091 (6)

### Geometric parameters (Å, °)

**Table:**

Ga1—O1	1.9354 (10)	Ga2—O5	1.9654 (11)
Ga1—O1^i^	1.9354 (10)	N1—O9	1.2311 (19)
Ga1—O3	1.9438 (10)	N1—O8	1.2385 (18)
Ga1—O3^i^	1.9438 (10)	N1—O7	1.2513 (18)
Ga1—O2	1.9515 (11)	N2—O13	1.2343 (16)
Ga1—O2^i^	1.9515 (11)	N2—O15	1.2418 (16)
Ga2—O4^ii^	1.9280 (10)	N2—O14	1.2660 (16)
Ga2—O4	1.9280 (10)	N3—O11	1.2407 (17)
Ga2—O6^ii^	1.9510 (12)	N3—O12	1.2435 (17)
Ga2—O6	1.9510 (12)	N3—O10	1.2533 (16)
Ga2—O5^ii^	1.9654 (11)		
			
O1—Ga1—O1^i^	180.0	O6^ii^—Ga2—O6	180.00 (8)
O1—Ga1—O3	89.45 (5)	O4^ii^—Ga2—O5^ii^	87.28 (5)
O1^i^—Ga1—O3	90.55 (5)	O4—Ga2—O5^ii^	92.72 (5)
O1—Ga1—O3^i^	90.55 (5)	O6^ii^—Ga2—O5^ii^	89.74 (6)
O1^i^—Ga1—O3^i^	89.45 (5)	O6—Ga2—O5^ii^	90.26 (6)
O3—Ga1—O3^i^	180.0	O4^ii^—Ga2—O5	92.72 (5)
O1—Ga1—O2	90.62 (5)	O4—Ga2—O5	87.28 (5)
O1^i^—Ga1—O2	89.38 (5)	O6^ii^—Ga2—O5	90.26 (6)
O3—Ga1—O2	89.23 (5)	O6—Ga2—O5	89.74 (6)
O3^i^—Ga1—O2	90.77 (5)	O5^ii^—Ga2—O5	180.0
O1—Ga1—O2^i^	89.38 (5)	O9—N1—O8	119.25 (15)
O1^i^—Ga1—O2^i^	90.62 (5)	O9—N1—O7	119.68 (14)
O3—Ga1—O2^i^	90.77 (5)	O8—N1—O7	121.06 (14)
O3^i^—Ga1—O2^i^	89.23 (5)	O13—N2—O15	121.45 (12)
O2—Ga1—O2^i^	179.999 (2)	O13—N2—O14	120.01 (12)
O4^ii^—Ga2—O4	180.0	O15—N2—O14	118.54 (12)
O4^ii^—Ga2—O6^ii^	88.81 (5)	O11—N3—O12	120.28 (13)
O4—Ga2—O6^ii^	91.19 (5)	O11—N3—O10	119.68 (13)
O4^ii^—Ga2—O6	91.19 (5)	O12—N3—O10	120.04 (12)
O4—Ga2—O6	88.81 (5)		

Symmetry codes: (i) −*x*+1, −*y*, −*z*; (ii) −*x*, −*y*, −*z*+1.

### Hydrogen-bond geometry (Å, °)

**Table:**

*D*—H···*A*	*D*—H	H···*A*	*D*···*A*	*D*—H···*A*
O18—H18···O8	0.80 (2)	2.26 (2)	2.9348 (18)	142 (2)
O16—H14···O18	0.83 (2)	2.07 (2)	2.8732 (19)	164 (2)
O5—H10···O7	0.82 (2)	1.91 (2)	2.7052 (17)	163 (2)
O1—H1···O16	0.81 (2)	1.85 (2)	2.6474 (16)	168 (2)
O4—H7···O14	0.81 (2)	1.83 (2)	2.6399 (15)	175 (2)
O5—H9···O17	0.81 (2)	1.87 (2)	2.676 (2)	174 (2)
O18—H17···O14	0.82 (2)	2.08 (2)	2.8729 (18)	163 (2)
O3—H6···O15^iii^	0.81 (2)	1.90 (2)	2.7150 (16)	175 (2)
O1—H2···O10^iii^	0.81 (2)	1.85 (2)	2.6545 (16)	175 (2)
O2—H4···O16^iii^	0.79 (2)	1.90 (2)	2.6895 (18)	175 (2)
O4—H8···O17^iv^	0.82 (2)	1.82 (2)	2.6312 (16)	171 (2)
O17—H15···O9^iv^	0.81 (2)	1.98 (2)	2.7791 (19)	171 (2)
O3—H5···O13^v^	0.79 (2)	1.96 (2)	2.7454 (16)	171 (2)
O6—H12···O12^vi^	0.80 (2)	1.93 (2)	2.7179 (16)	174 (2)
O16—H13···O18^vii^	0.82 (2)	1.93 (2)	2.7525 (19)	177 (3)
O6—H11···O11^viii^	0.80 (2)	1.90 (2)	2.6938 (17)	176 (2)
O2—H3···O8^i^	0.79 (2)	1.94 (2)	2.7269 (17)	169 (2)
O17—H16···O7^ix^	0.80 (2)	2.03 (2)	2.7675 (18)	154 (2)

Symmetry codes: (iii) *x*, −*y*+1/2, *z*−1/2; (iv) *x*, −*y*+1/2, *z*+1/2; (v) *x*, *y*, *z*−1; (vi) *x*−1, −*y*+1/2, *z*−1/2; (vii) −*x*+1, −*y*, −*z*+1; (viii) *x*−1, *y*, *z*; (i) −*x*+1, −*y*, −*z*; (ix) −*x*, *y*+1/2, −*z*+1/2.

## References

[bb1] Bruker (2008). *APEX2*, *SAINT* and *SADABS* Bruker AXS Inc., Madison, Wisconsin, USA.

[bb2] Farrugia, L. J. (1997). *J. Appl. Cryst.* **30**, 565.

[bb3] Hair, N. J. & Beattie, J. K. (1977). *Inorg. Chem.* **16**, 245–250.

[bb4] Lazar, D., Ribár, B., Divjaković, V. & Mészáros, Cs. (1991). *Acta Cryst.* C**47**, 1060–1062.

[bb5] Lazar, D., Ribár, B. & Prelesnik, B. (1991). *Acta Cryst.* C**47**, 2282–2285.

[bb6] Rudolph, W. W., Pye, C. C. & Irmer, G. (2002). *J. Raman Spectrosc.* **33**, 177–190.

[bb7] Shannon, R. D. & Prewitt, C. T. (1969). *Acta Cryst.* B**25**, 925–946.

[bb8] Sheldrick, G. M. (2008). *Acta Cryst.* A**64**, 112–122. 10.1107/S0108767307043930 18156677

